# Comprehensive analysis of angiogenesis and stemness-related genes in chemotherapy and immunotherapy of bladder cancer

**DOI:** 10.17305/bb.2025.12046

**Published:** 2025-04-18

**Authors:** Zhixiang Yin, Yifan Qiu, Chenfei Xu, Changsong Pei

**Affiliations:** 1 Urology Ward, The First Affiliated Hospital of Soochow University, Suzhou, Jiangsu, China; 2 Urology Ward, Jiangsu Province Geriatric Hospital, Nanjing, Jiangsu, China

**Keywords:** Prognosis, angiogenesis, stemness, chemotherapy, immunotherapy

## Abstract

Tumor angiogenesis and cancer stem cells (CSCs) are critical features of malignancies. Research has shown that CSCs promote blood vessel formation, while increased vasculature, in turn, supports CSC proliferation—creating a detrimental feedback loop that drives disease progression. However, studies investigating vascularization and stem-like properties in bladder cancer (BLCA) remain limited. In our investigation, we applied clustering techniques and LASSO methodology to assess the significance of vascularization- and stemness-related genes in predicting responses to chemotherapy and immunotherapy in BLCA. Using multivariate Cox regression analysis, we identified Von Hippel–Lindau (VHL) as the primary prognostic marker associated with both vascularization and stem-like traits. Tissue array analysis of 40 BLCA specimens, combined with molecular docking simulations, revealed interactions between HDAC6 and VHL that influence stem-like behavior and angiogenesis in BLCA. Additionally, VHL showed strong correlations with treatment responses to both chemotherapy and immunotherapy in BLCA. In conclusion, our findings highlight the critical role of vascularization- and stemness-related genes in determining therapeutic outcomes in BLCA and underscore the regulatory relationship between VHL and HDAC6 in modulating treatment response.

## Introduction

Bladder cancer (BLCA), a malignant tumor arising from the bladder’s mucous membrane, is one of the deadliest cancers of the urinary tract [1]. Currently, non-muscle invasive BLCA (NMIBC) accounts for approximately 75% of diagnosed cases and is generally responsive to chemotherapy and surgical resection. However, about 20% of NMIBC cases eventually progress to muscle-invasive BLCA (MIBC). The standard treatment for MIBC includes radical cystectomy with pelvic lymph node dissection [2]. Despite this aggressive approach, the five-year survival rate remains suboptimal. For advanced uroepithelial carcinoma, gemcitabine, and cisplatin are commonly used as first-line therapies [3]. Additionally, immune checkpoint inhibitors (ICIs) targeting PD-1/PD-L1 have shown promise in treating patients with platinum-refractory disease. Nevertheless, fewer than half of patients with advanced BLCA benefit from ICIs, despite encouraging results and progress in various treatment strategies [4]. Given the high recurrence and mortality rates, there remains an urgent need to explore curative mechanisms and identify novel therapeutic targets for BLCA [5]. In 1970, Dr. Judah Folkman proposed that tumor growth and metastasis depend on angiogenesis, suggesting that inhibiting this process could be an effective therapeutic strategy [6]. Over recent decades, targeting angiogenic mediators has become a key focus in cancer treatment and prevention. Vascular endothelial growth factors (VEGFs) are pivotal in tumor-associated angiogenesis, and VEGF inhibition has demonstrated therapeutic benefits across multiple cancer types [7, 8]. For instance, in gastric cancer, VEGF upregulation promotes chemoresistance to oxaliplatin via enhanced angiogenesis [9], whereas in hepatocellular carcinoma, VEGF downregulation improves sensitivity to sorafenib [10]. Cancer stem cells (CSCs) are a distinct tumor subpopulation defined by their self-renewal, continuous proliferative potential, and multipotent differentiation [11, 12]. Although they represent a small proportion of the tumor, CSCs play a major role in tumor progression, aggressiveness, invasion, therapeutic resistance, metastasis, and recurrence [13]. For example, in gastric cancer, WNT2-SOX4 feedback loops drive drug resistance and tumor growth by enhancing stem-like properties [14], while in breast cancer, CSC expansion facilitates metastasis [15]. Emerging research highlights the synergistic relationship between tumor vasculature and CSCs in promoting cancer development. CSCs contribute to vessel formation by secreting angiogenic factors and, in some cases, differentiating into vascular endothelial cells (ECs) [16]. Meanwhile, tumor vasculature supplies CSCs with oxygen, nutrients, and essential maintenance signals, creating a supportive niche [17]. This reciprocal interaction forms a self-sustaining loop that plays a critical role in tumor progression. In this study, we investigated angiogenesis- and stemness-related genes with differential expression between BLCA tissues and adjacent normal tissues, analyzing their associations with patient outcomes. Using subgroup classification and Least Absolute Shrinkage and Selection Operator (LASSO) analysis, we explored gene expression patterns, survival correlations, immune cell infiltration, and predicted drug responses in BLCA. Our results identified Von Hippel (VHL) as the most significant prognostic marker among the angiogenesis- and stemness-associated genes, highlighting its relevance in the immunotherapeutic landscape of BLCA. Additionally, we uncovered a novel association between VHL and HDAC6 in BLCA. Collectively, our findings position VHL as a key player in BLCA progression, offering potential as both a therapeutic target and prognostic biomarker, particularly in relation to chemotherapy sensitivity and immunotherapy responsiveness.

## Materials and methods

### Data acquisition

We collected 2015 genes related to angiogenesis and 14,645 genes associated with stemness from GeneCards (score >0.7). Transcriptional data and patient information for 406 BLCA cases and 19 normal bladder specimens were obtained from The Cancer Genome Atlas (TCGA, https://portal.gdc.cancer.gov/). Differential expression analysis between tumor and normal tissues was conducted using the Benjamini–Hochberg false discovery rate (FDR) correction (FDR < 0.05). For validation, we used data from the Gene Expression Omnibus (GEO, https://www.ncbi.nlm.nih.gov/geo/), comprising 165 primary BLCA samples and nine normal bladder specimens. Additionally, we acquired BLCA tissue microarrays from Shanghai Outdo Biotech Company, which included 40 bladder samples. The investigation was approved by the Ethics Committee of Shanghai Outdo Biotech Company.

### Mutation frequency, somatic copy number and pathway activity analysis

The occurrence of alterations in genes associated with angiogenesis and stemness was evaluated using the Gene Set Cancer Analysis (GSCA, http://bioinfo.life.hust.edu.cn/GSCA/#/) datasets. This analysis included data on single nucleotide variants (SNVs), gene amplifications, and both complete and heterozygous deletions [18]. GSCA also performed correlation analyses of established cancer-related pathways, with the strength of associations measured using Spearman’s correlation.

### Consistency cluster analysis

Consistency analysis was performed using the ConsensusClusterPlus R package (v1.54.0). Sampling 80% of the total cases was repeated 100 times, allowing for the formation of up to six distinct clusters. Hierarchical clustering was applied with clusterAlg ═ “hc” and innerLinkage ═ “ward.D2.”

### Development of prognostic models

Twelve angiogenesis- and stemness-related prognostic genes were identified, and a prognostic model was constructed using the TCGA-BLCA dataset via the LASSO method. The model was validated using the GSE13507 dataset [19]. Feature selection was performed using LASSO regression with 10-fold cross-validation [20], implemented through the glmnet package in R.

### Functional enrichment analysis

To further confirm the potential roles of angiogenesis- and stemness-related genes, a functional enrichment analysis was performed. The Kyoto Encyclopedia of Genes and Genomes (KEGG) database was used to explore gene functions and obtain comprehensive genomic insights. Additionally, Gene Set Enrichment Analysis (GSEA) was conducted using the clusterProfiler package.

### Immune infiltration and chemotherapeutic drug sensitivity analysis

To evaluate the immune scores of angiogenesis- and stemness-related genes in BLCA, we used immunedeconv, an R package that integrates six advanced algorithms: TIMER, xCell, MCP-counter, CIBERSORT, EPIC, and quanTIseq. For our analysis, we specifically implemented the xCell algorithm, as it effectively assesses a wide range of immune cell populations—making it well-suited for our research objectives. To predict the potential immune checkpoint blockade (ICB) response, we applied the TIDE algorithm. Additionally, chemotherapy response was estimated for each specimen using data from the Genomics of Drug Sensitivity in Cancer (GDSC, https://www.cancerrxgene.org/). This prediction process was conducted using the R package pRRophetic.

### Prediction of ubiquitinated gene target proteins and analysis of protein interactions

Target gene prediction for the E3 ubiquitin ligase VHL was performed using the UbiBrowser 2.0 website [21, 22]. Protein–protein docking was carried out using the ZDOCK algorithm to investigate the interaction between VHL and HDAC6. The PDB files for the protein domains were obtained from the Protein Data Bank (http://www.rcsb.org/). ZDOCK was used to identify docking sites and calculate the corresponding docking scores.

### Immunohistochemical staining analysis of VHL and HDAC6 expression in BLCA

Tissue microarrays underwent multiple preparation steps. The process began with heating in an oven at 85 ^∘^C for 10 min. The arrays were then submerged in xylene for 15 min and rehydrated through a series of descending ethanol concentrations (100%, 95%, 80%, and 70%). Antigen retrieval was performed using a citric acid solution in an autoclave. After cooling to room temperature, the samples were washed with PBS and treated with hydrogen peroxide for 20 min. Next, primary antibodies were applied: VHL (1:200 dilution, Catalog No. 24756-1-AP, Proteintech, Wuhan, China) and HDAC6 (1:100 dilution, Catalog No. 12834-1-AP, Proteintech, Wuhan, China). The arrays were incubated at room temperature for 2 h. This was followed by three PBS washes and a 20-min incubation with an immunohistochemical secondary antibody at room temperature. After three additional PBS rinses, DAB staining was performed, followed by hematoxylin counterstaining. The arrays were then dehydrated through ascending ethanol concentrations (70%, 80%, 90%, and 100%) and immersed in xylene for 8 min. Finally, microarray blocking was conducted. Immunostaining was evaluated using intensity scores ranging from 0 to –3, where 0 indicated no reaction, 1 indicated a mild reaction, 2 indicated a moderate reaction, and 3 indicated a strong reaction. Distribution scores were assigned as follows: 1 (0%–25%), 2 (26%–50%), 3 (51%–75%), and 4 (76%–100%). The final score was calculated by multiplying the intensity and distribution scores.

### Statistical analysis

All analytical procedures were conducted using R version 4.0.3 (R Foundation for Statistical Computing, Vienna, Austria) and GraphPad Prism 8.0 (GraphPad Software, San Diego, CA, USA). The normality of data distribution was assessed using the Shapiro–Wilk test. Continuous variables are presented as mean ± standard deviation (SD) for normally distributed data, or as median (interquartile range; P25, P75) for non-normally distributed data. Comparisons between two independent groups were performed using the independent Student’s *t*-test for normally distributed data, or the Mann–Whitney *U* test for non-normally distributed data. Associations between variables were evaluated using Spearman’s correlation. The accuracy of prognostic models was assessed with ROC curves and AUC, calculated via the trapezoidal rule. Survival outcomes were analyzed using Kaplan–Meier curves and the log-rank test. Independent prognostic factors were identified through multivariate Cox proportional hazards regression. Statistical significance was defined as *P* < 0.05. Significance levels were denoted as follows: *P < 0.05, **P < 0.01, ***P < 0.001.

## Results

### Examination of angiogenesis and stemness-related genes in BLCA

**Figure 1.**
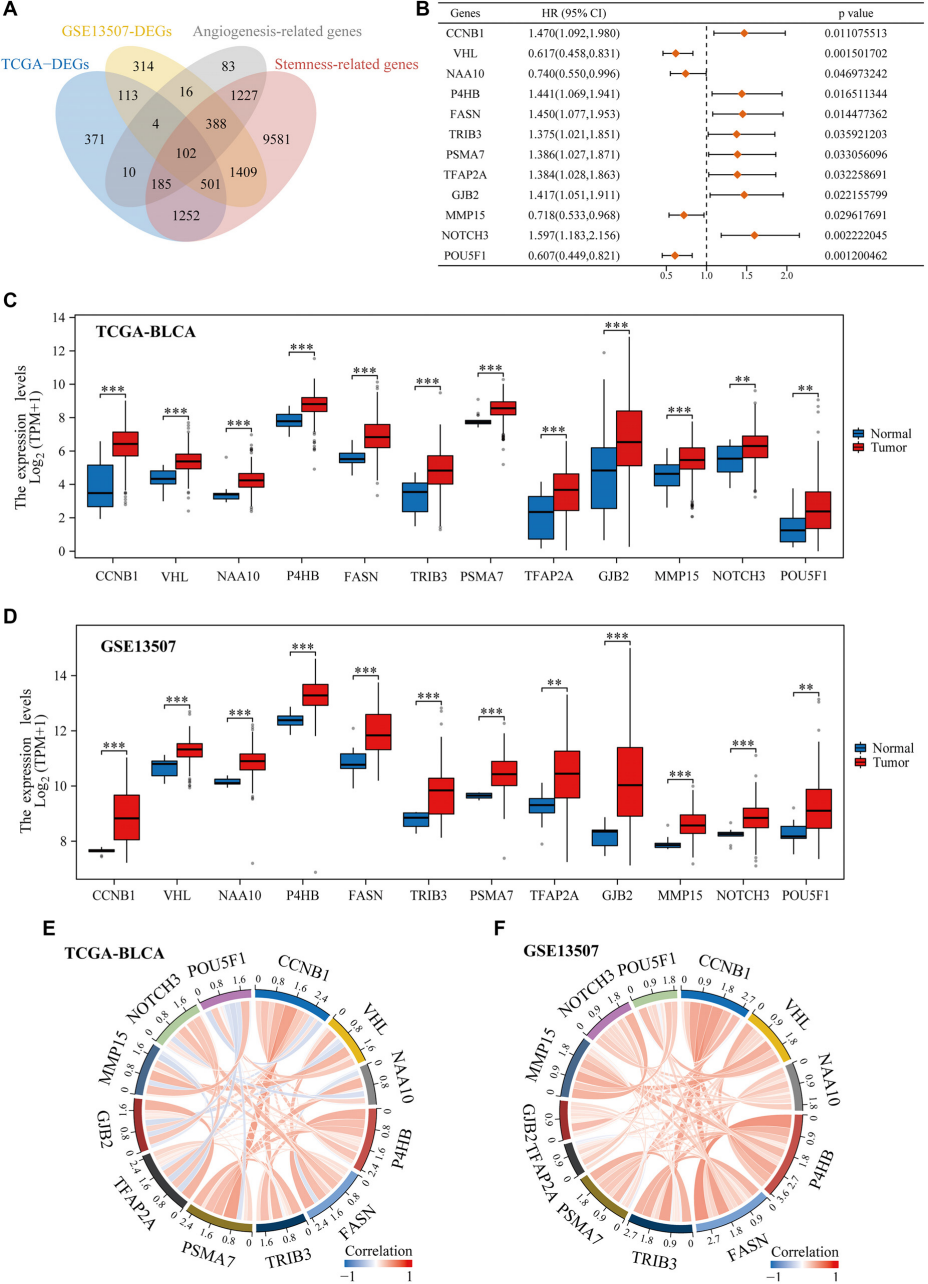
**Recognition of differential prognostic genes linked to angiogenesis and stemness in BLCA.** (A) Venn diagram depicting the overlap of genes linked to angiogenesis and stemness; (B) Forest plot illustrating survival differences among 12 identified genes; (C) Gene expression patterns of angiogenesis- and stemness-associated differential prognostic markers within TCGA-BLCA dataset; (D) Gene expression profiles of angiogenic and stemness-related differential prognostic indicators in the GSE13507 dataset; (E) Analysis of relationships between angiogenesis and stemness-related differential prognostic markers in TCGA-BLCA dataset; (F) Assessment of relationships between angiogenesis and stemness-related differential prognostic indicators in the GSE13507 dataset. TCGA: The Cancer Genome Atlas; BLCA: Bladder cancer; VHL: Von Hippel–Lindau.

To investigate the significance of genes associated with angiogenesis and stemness in BLCA, we first retrieved relevant genes from GeneCards. We then intersected these with the upregulated differentially expressed genes identified in both the TCGA-BLCA and GSE13507 datasets, resulting in 102 genes associated with stemness and angiogenesis (Figure 1A). Among these, 12 genes were found to be significantly associated with prognosis (Figure 1B). The expression patterns of these 12 genes in tumor vs normal tissues from the TCGA-BLCA and GSE13507 datasets were visualized using boxplots (Figure 1C and 1D). Furthermore, a correlation analysis revealed that most of these genes were positively correlated with each other in both datasets (Figure 1E and 1F).

### Functional analysis and genetic alterations in angiogenesis and stemness-related differential prognostic genes

**Figure 2.**
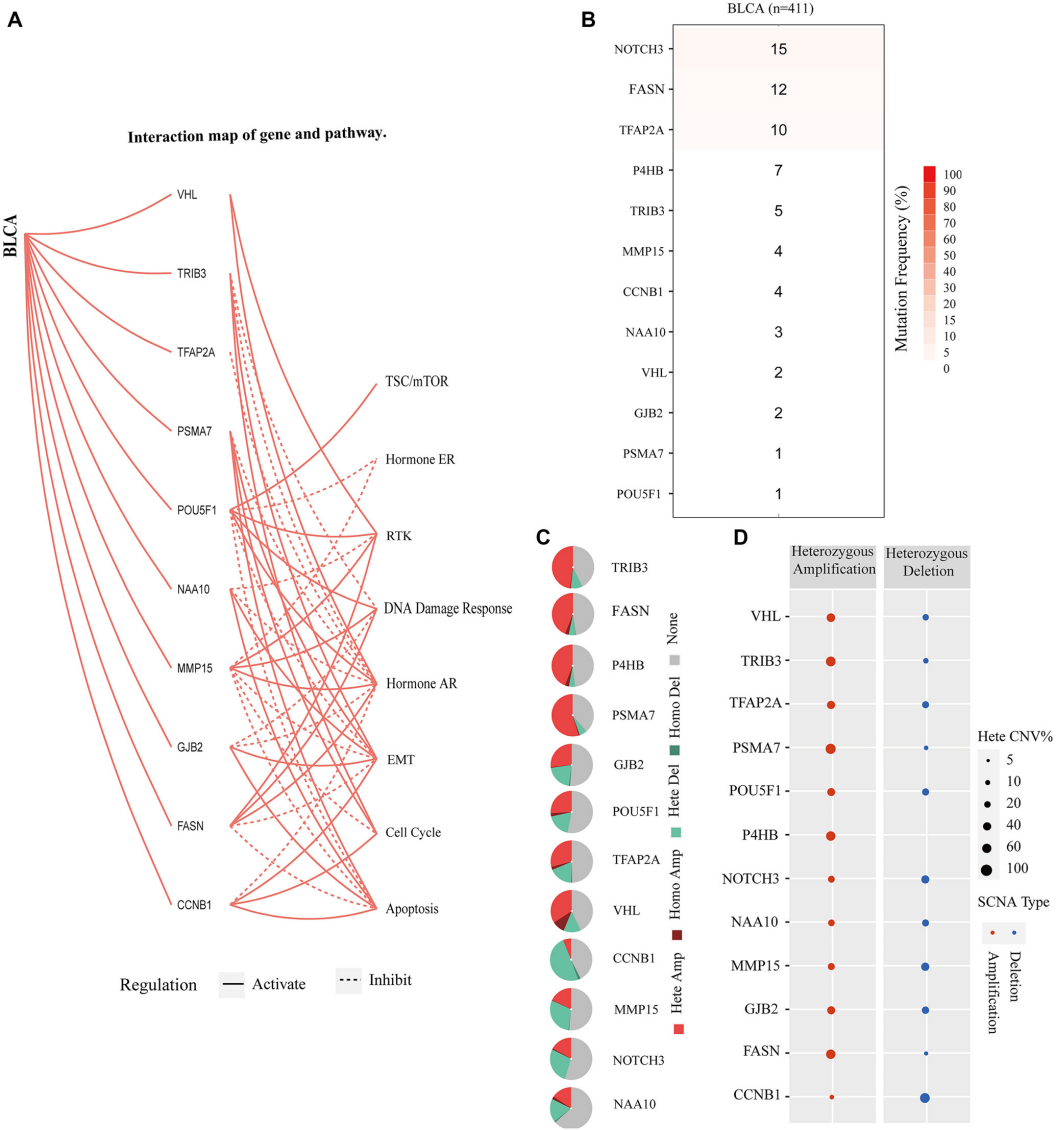
**Functional network and genetic modifications of angiogenesis and stemness-related genes in BLCA.** (A) Roles of blood vessel formation and stem cell-associated genes; (B) Analysis of mutation rates for blood vessel formation and stem cell-associated genes in BLCA; (C) The copy number variation data of blood vessel formation and stem cell-associated genes in BLCA; (D) The proportion of heterozygous copy number variation for blood vessel formation and stem cell-associated genes in BLCA. TCGA: The Cancer Genome Atlas; BLCA: Bladder cancer; VHL: Von Hippel–Lindau.

To examine the mutation profiles of genes involved in angiogenesis and stemness across various cancer types, we used GSCALite to assess relevant pathways, SNVs, and CNVs in 12 key genes, with a particular focus on BLCA. The functional network analysis revealed several regulatory relationships in BLCA: the TSC/mTOR pathway can be positively regulated by POU5F1; hormone ER signaling can be negatively regulated by POU5F1 and MMP15; RTK signaling can be positively regulated by VHL, POU5F1, MMP15, and FASN, and negatively regulated by NAA10. The DNA damage response appears to be negatively regulated by TRIB3, while GJB2 is negatively regulated by POU5F1 and MMP15. FASN is positively regulated, and hormone AR signaling is positively regulated by POU5F1, FASN, VHL, and MMP15, but negatively regulated by other genes. EMT is positively regulated by CCNB1, GJB2, NAA10, TRIB3, and PSMA7, and negatively regulated by other genes. The cell cycle is positively regulated by CCNB1, PSMA7, and TRIB3, and negatively regulated by MMP15. Apoptosis is positively regulated by CCNB1, GJB2, NAA10, and PSMA7, and negatively regulated by FASN, MMP15, and POU5F1 (Figure 2A). Among these genes, NOTCH3 exhibited the highest frequency of harmful mutations in BLCA based on the SNV percentage heatmap (Figure 2B). Additionally, we analyzed the distribution of CNV types—including heterozygous amplification, heterozygous deletion, homozygous amplification, and homozygous deletion—among angiogenesis- and stemness-associated genes in BLCA patients (Figure 2C). Our findings further showed that gene expression levels associated with angiogenesis and stemness were positively correlated with heterozygous amplification CNVs, and negatively correlated with heterozygous deletion CNVs (Figure 2D).

### Cluster analysis of angiogenesis and stemness-related genes in BLCA

**Figure 3.**
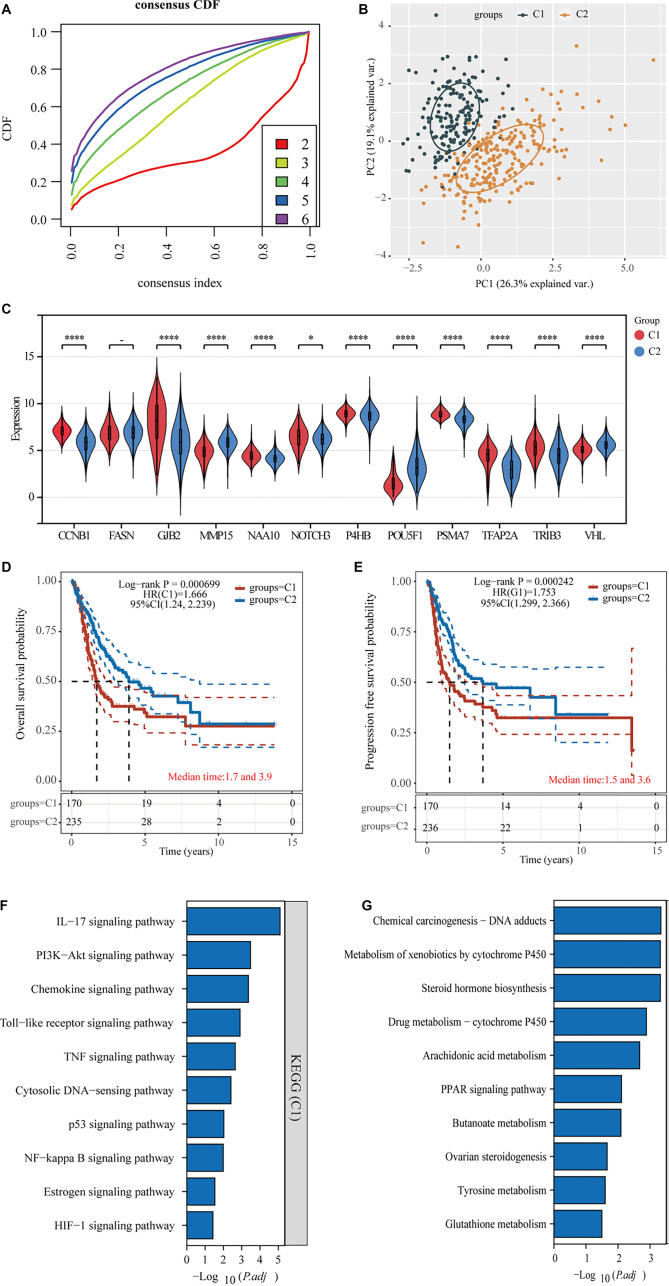
**Subgroup typing of angiogenesis and stemness-related genes.** (A) CDF curve clustering evaluation dividing angiogenesis- and stemness-associated genes into 2–6 distinct cohorts; (B) Distribution of specimens across the two identified cohorts; (C) Comparative gene expression analysis of angiogenesis and stemness markers between both cohorts; (D) Overall survival assessment using KM plots comparing the two cohorts; (E) Disease-free survival evaluation through KM analysis between both cohorts; (F and G) Functional enrichment investigation of elevated genes across the two cohorts. VHL: Von Hippel–Lindau.

To examine the roles of angiogenesis- and stemness-associated genes in BLCA, we performed subgroup classification based on these key genes [23]. Evaluation of the CDF curve indicated that dividing the samples into two clusters provided the most appropriate grouping (Figure 3A). PCA effectively visualized the distribution of samples across these two clusters (Figure 3B). In terms of gene expression, MMP15, POU5F1, and VHL were more highly expressed in cluster 2 compared to cluster 1, while the remaining genes showed increased expression in cluster 1 (Figure 3C). We also compared survival outcomes between the two clusters and found that cluster 1 was associated with poorer overall and disease-free survival rates than cluster 2 (Figure 3D and 3E). To explore the mechanisms underlying this prognostic difference among BLCA patients, we conducted a KEGG enrichment analysis. The upregulated genes in cluster 1 were significantly enriched in several cancer-related pathways, including the IL-17 signaling pathway, the PI3K-Akt signaling pathway, and the TNF signaling pathway. In contrast, the upregulated genes in cluster 2 were primarily associated with the PPAR signaling pathway (Figure 3F and 3G).

### Examination of angiogenesis and stem cell-related genes with immune infiltration and drug sensitivity in BLCA

**Figure 4.**
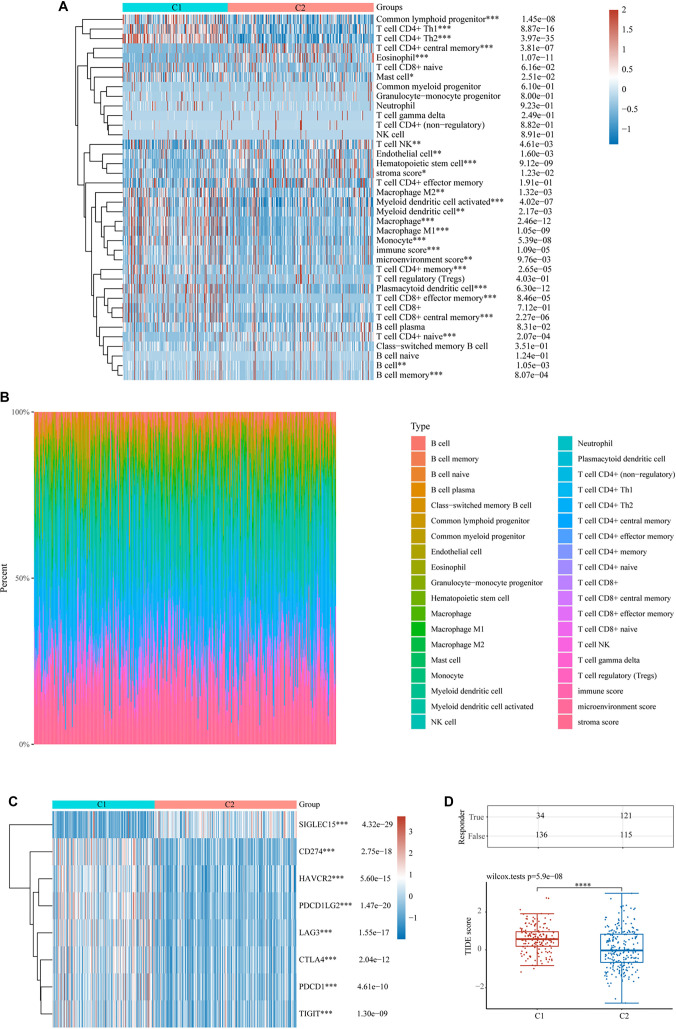
**Immune microenvironment in BLCA shows a substantial correlation with genes implicated in angiogenesis and stemness.** (A) Visual representation of immune cell score patterns; (B) Proportional distribution of infiltrating immune cells across individual specimens; (C) Expression profile matrix of genes linked to immune checkpoint regulation; (D) Upper section: Quantitative analysis of immune responses among distinct cohorts in the predicted outcomes; lower section: Immune response score variations across different categories in the predicted findings. BLCA: Bladder cancer.

**Figure S1.**
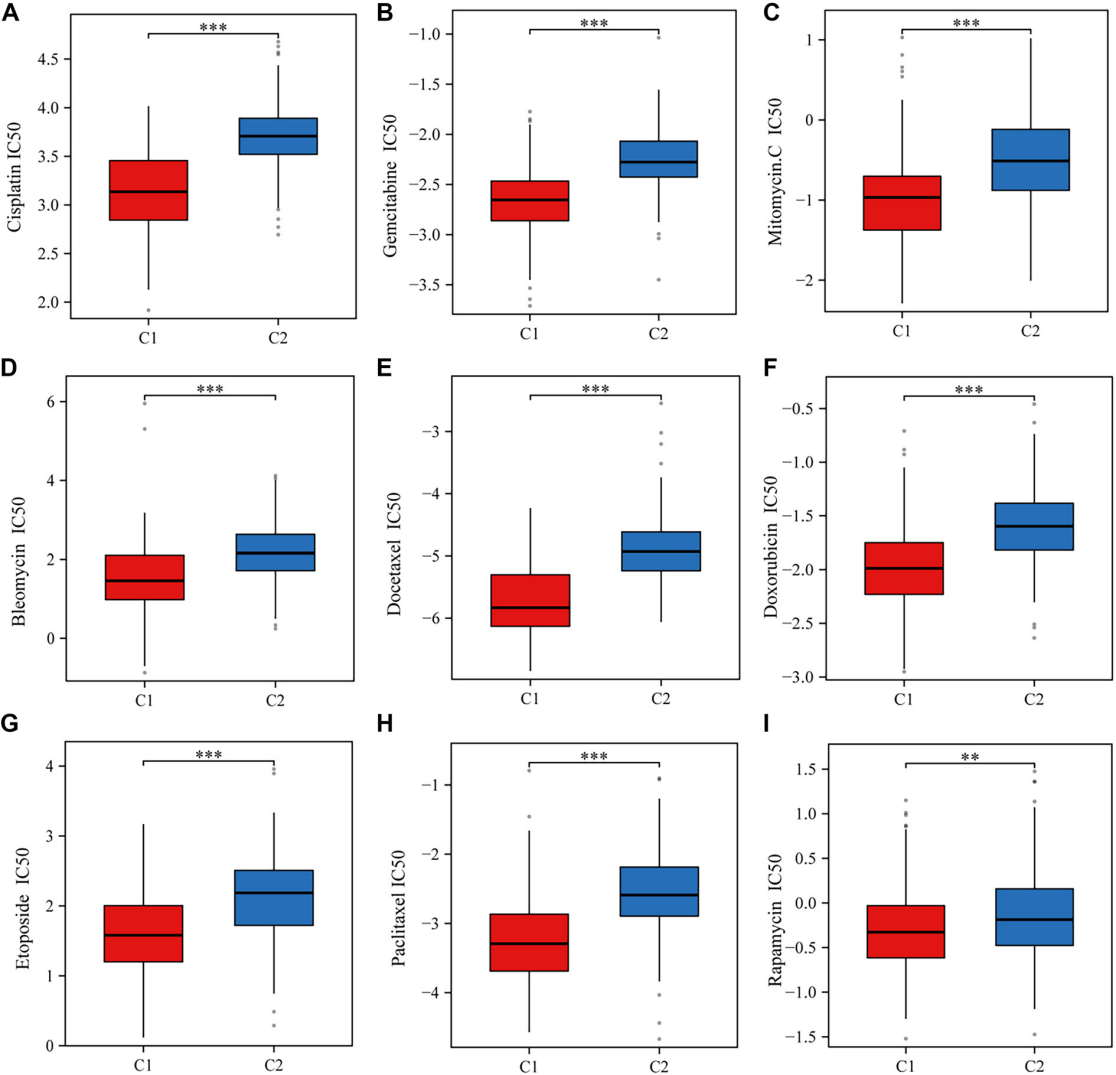
**IC50 values for nine chemotherapy drugs were significantly lower in Cluster 1 compared to Cluster 2, suggesting that angiogenesis- and stemness-related genes influence chemotherapy sensitivity in BLCA.** BLCA: Bladder cancer.

xCell was used to assess various immune cell populations and examine the relationship between angiogenesis, stemness-associated genes, and immune infiltration in BLCA. Significant differences in immune cell populations were observed between the two clusters, including Common Lymphoid Progenitors, CD4+ Th1 and Th2 T Cells, CD4+ Central Memory T Cells, Eosinophils, Mast Cells, NK T Cells, ECs, Hematopoietic Stem Cells, and Stroma Score. Additional variations were noted in Macrophage M2, Activated and General Myeloid Dendritic Cells, Macrophages (including M1), Monocytes, Immune Score, Microenvironment Score, CD4+ Memory and Naive T Cells, Plasmacytoid Dendritic Cells, and CD8+ Effector and Central Memory T Cells (Figure 4A). The distribution of these infiltrating immune cells in BLCA specimens is shown in Figure 4B. Furthermore, immune checkpoint-related gene expression varied significantly between clusters (Figure 4C), indicating a strong correlation between immune infiltration in BLCA and genes associated with angiogenesis and stemness. TIDE analysis was employed to assess two immune evasion mechanisms: impaired cytotoxic T lymphocyte (CTL) function and CTL exclusion due to immunosuppressive factors. Higher TIDE scores predict lower effectiveness of ICB therapy and worse survival outcomes post-ICB. Cluster 1 exhibited significantly higher TIDE scores than cluster 2, suggesting reduced survival following ICB treatment in cluster 1 patients (Figure 4D). Common chemotherapy agents for BLCA include Cisplatin, Gemcitabine, Mitomycin C, Bleomycin, Docetaxel, Doxorubicin, Etoposide, Paclitaxel, and Rapamycin. We investigated potential associations between angiogenesis- and stemness-related genes and sensitivity to these drugs. IC50 scores for all nine drugs were significantly lower in cluster 1 than in cluster 2 (Figure S1A–S1I), suggesting that genes linked to angiogenesis and stemness may influence drug sensitivity.

### Prognostic modeling based on angiogenesis and stemness-related genes

**Figure 5.**
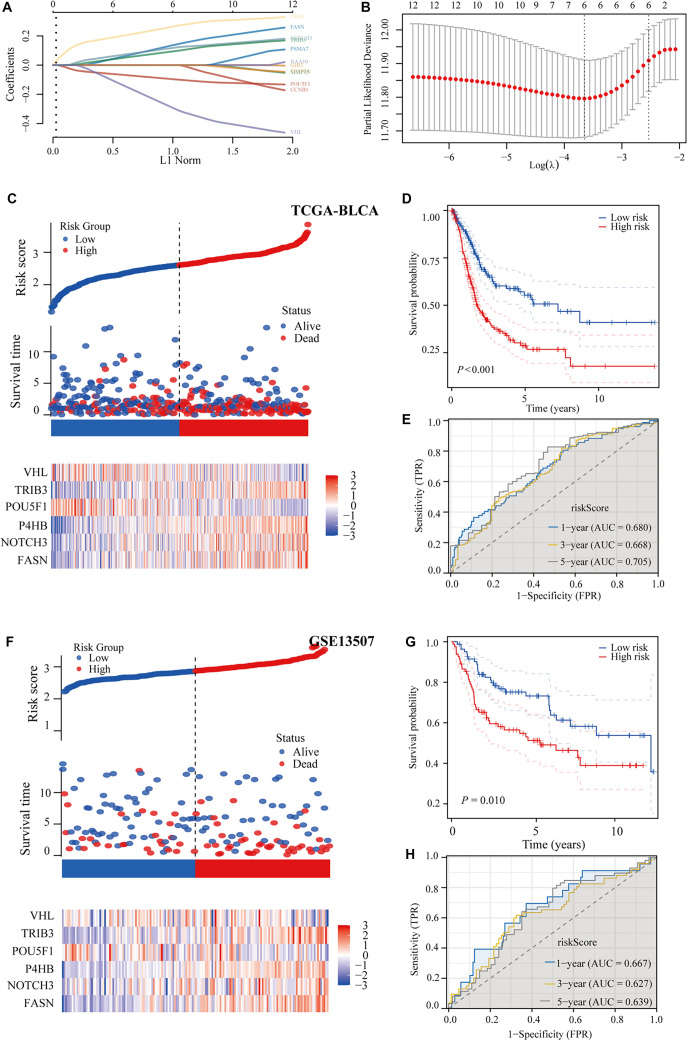
**Development and verification of predictive frameworks for angiogenesis and stemness.** (A and B) LASSO methodology selected the most significant prognostic indicators; (C–E) Establishment of survival prediction frameworks associated with angiogenesis and stemness-related using TCGA-BLCA cohort data; (F–H) Implementation of angiogenesis and stemness-related survival prediction frameworks within the GSE13507 cohort. TCGA: The Cancer Genome Atlas; BLCA: Bladder cancer; VHL: Von Hippel–Lindau; LASSO: Least Absolute Shrinkage and Selection Operator.

To identify the most critical angiogenesis- and stemness-associated prognostic genes in BLCA, we conducted a streamlined analysis using the LASSO technique on 12 candidate genes. The resulting predictive model incorporated six genes: VHL, TRIB3, POU5F1, P4HB, NOTCH3, and FASN (Figure 5A and 5B). The risk score was calculated using the following formula: Risk score ═ (0.1348 × FASN) + (0.1169 × NOTCH3) + (0.262 × P4HB) + (−0.0957 × POU5F1) + (0.0932 × TRIB3) + (−0.2896 × VHL). A heatmap was generated to illustrate the relationships among the risk score, survival duration, survival outcome, and the expression levels of the six selected genes in the TCGA-BLCA cohort (Figure 5C). The analysis showed that patients classified in the high-risk group had significantly shorter overall survival than those in the low-risk group (Figure 5D). ROC analysis confirmed the model’s predictive power for 1-, 3-, and 5-year survival outcomes, with particularly strong performance at the 5-year mark (Figure 5E). The model’s AUC values were 0.680, 0.668, and 0.705 for predicting one-, three-, and five-year survival, respectively. External validation using the GSE13507 dataset further supported the model’s robustness, yielding consistent results with those from the TCGA-BLCA cohort (Figure 5F–5H).

### Analysis of angiogenesis and stemness prognostic models correlating with immune infiltration and drug sensitivity in BLCA

**Figure 6.**
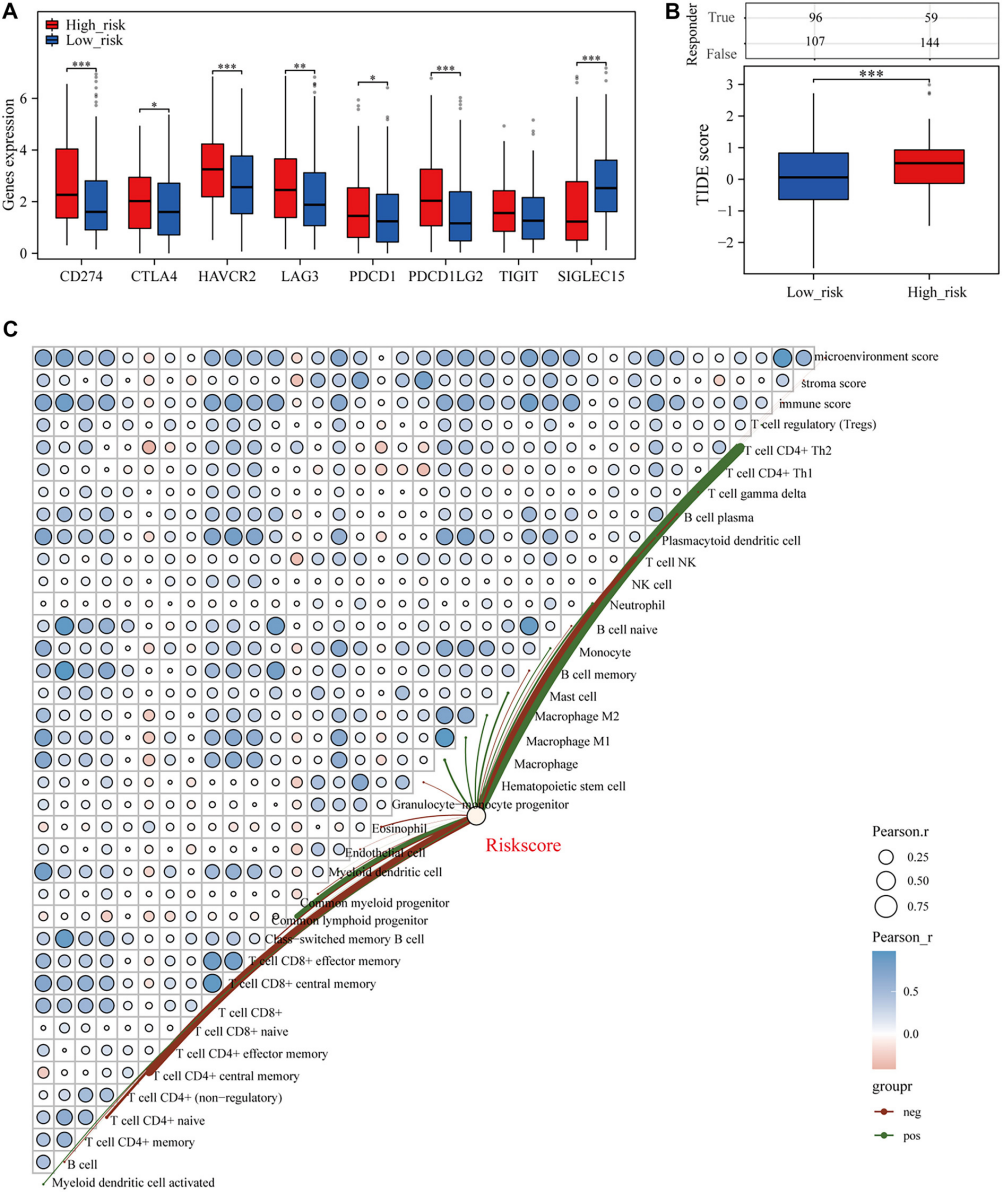
**Prognostic models were notably linked to the immune microenvironment of BLCA.** (A) Expression variations of immune checkpoint genes between elevated and reduced risk categories; (B) TIDE algorithm evaluates the potential response patterns of distinct risk cohorts to anticipated ICIs; (C) Examination of the link between risk assessment scores and immune-infiltrating cell populations utilizing the xCELL approach. BLCA: Bladder cancer; ICI: Immune checkpoint inhibitor.

**Figure S2.**
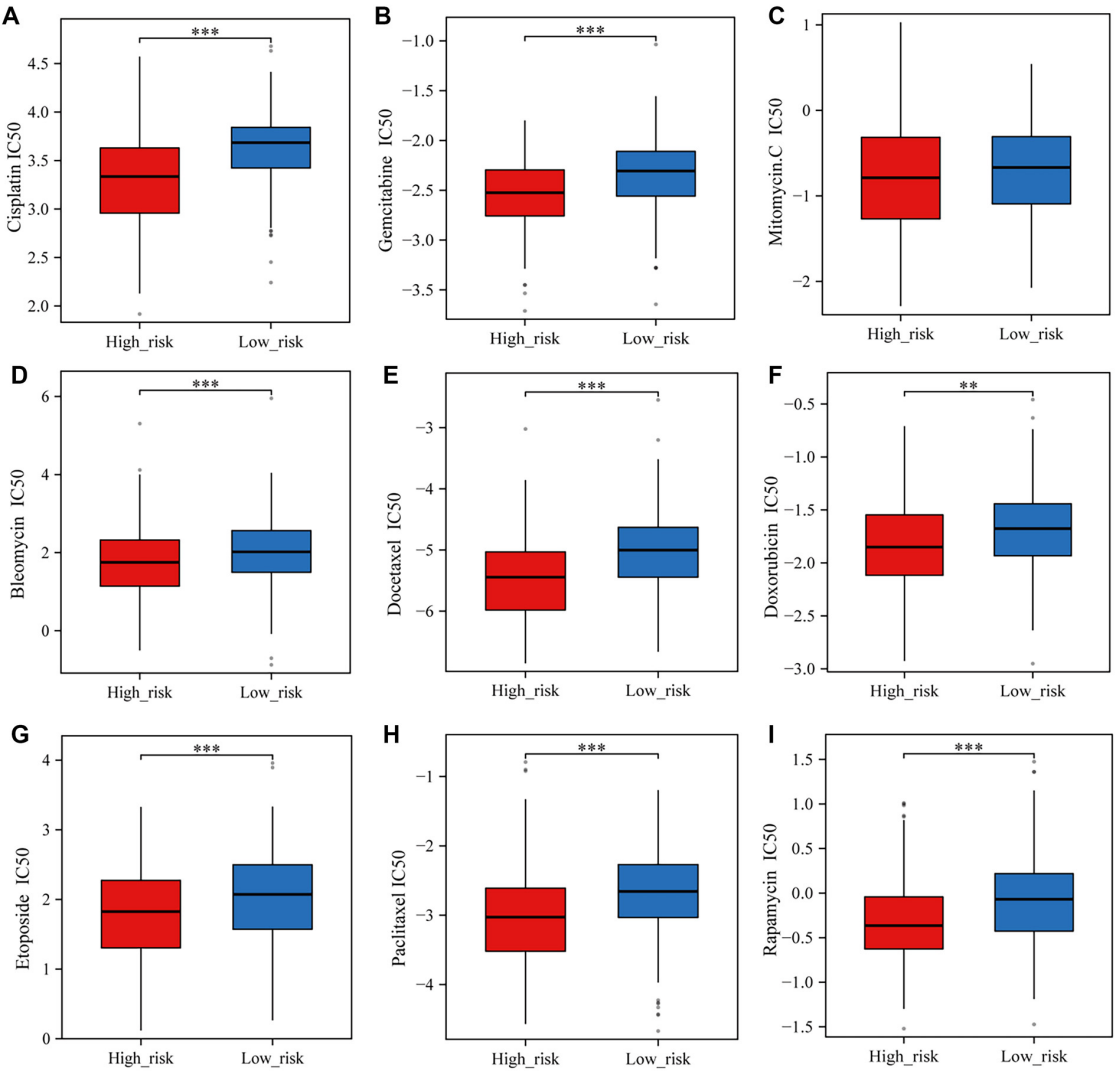
**The high-risk cohort showed lower IC50 values for all nine chemotherapy drugs, indicating that the prognostic model is associated with chemotherapy sensitivity in BLCA.** BLCA: Bladder cancer.

We investigated the relationship between our prognostic model—developed using the LASSO algorithm and integrating angiogenesis and stemness—and the immune microenvironment of BLCA. The analysis revealed significant differences in immune checkpoint gene expression between high-risk and low-risk cohorts. Specifically, SIGLEC15 was more highly expressed in the low-risk group, whereas other immune checkpoint-related genes were upregulated in the high-risk group (Figure 6A). Additionally, the high-risk cohort exhibited a higher TIDE score than the low-risk cohort, indicating a potentially shorter survival time following ICB therapy (Figure 6B). Using the xCell algorithm, we assessed the association between risk scores and immune infiltration-related cell types (Figure 6C). We also examined potential links between the angiogenesis- and stemness-based prognostic model and drug response. The high-risk group showed significantly lower IC50 values for all nine evaluated drugs compared to the low-risk group (Figure S2A–S2I), suggesting a connection between the model and increased drug sensitivity.

### VHL as the best angiogenesis and stemness-related prognostic gene in BLCA

**Figure 7.**
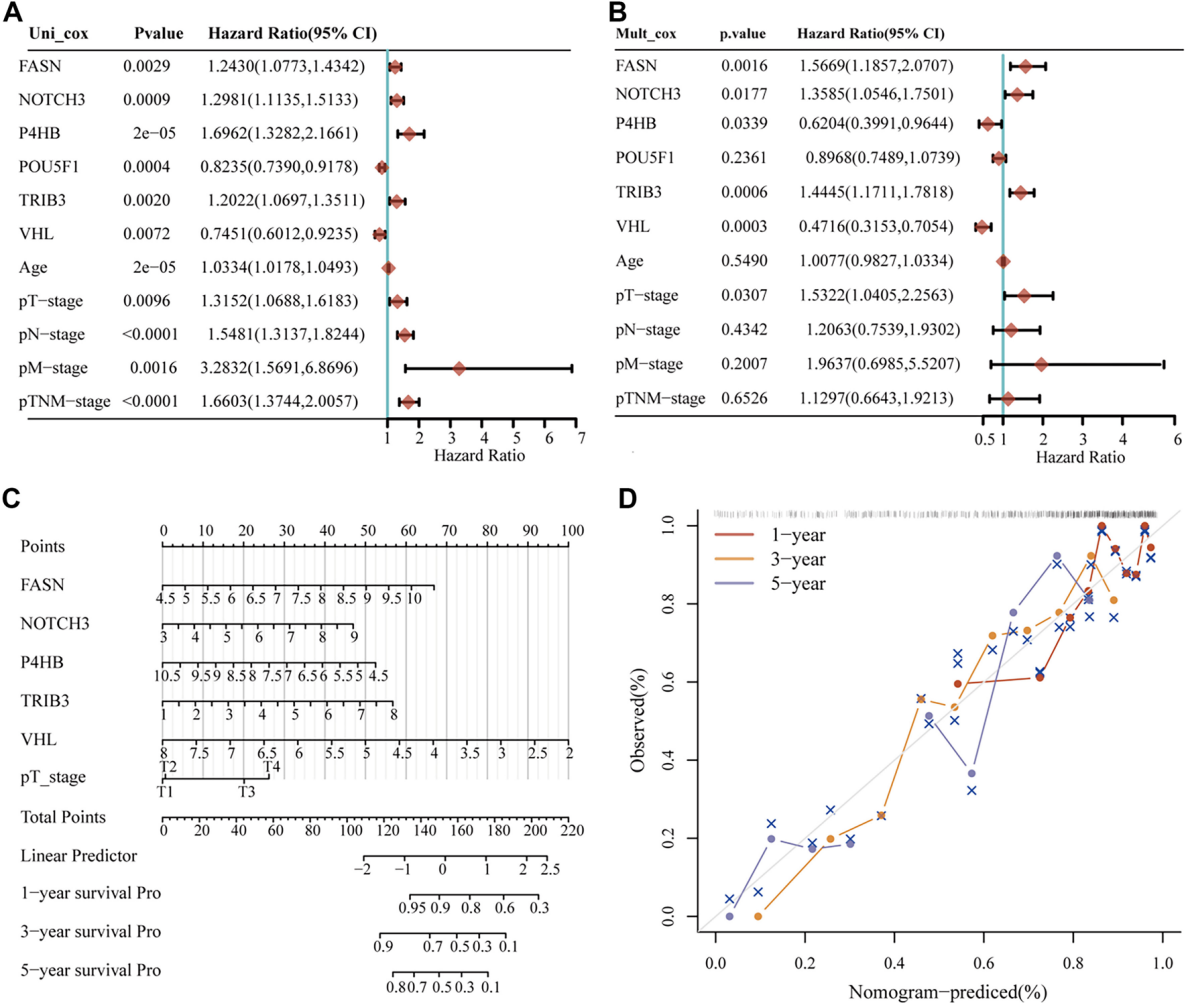
**Cox regression analysis of angiogenesis and stemness-related prognostic genes.** (A) Univariate Cox regression evaluation of survival significance for angiogenesis and stemness-linked genes; (B) Multivariate Cox regression assessment of survival indicators among angiogenesis and stemness-related genes; (C) Bar graph visualization derived from multivariate Cox findings; (D) Validation curves generated from multivariate Cox outcomes. VHL: Von Hippel–Lindau.

Drawing from the earlier subgroup classifications and established predictive framework analyses, research indicates that genes associated with angiogenesis and stemness show strong correlations with survival outcomes, immune cell infiltration, and chemotherapy response in BLCA patients. For the six genes included in the predictive framework, further evaluation incorporating clinical parameters is planned. Using the TCGA-BLCA database, we performed a multifactor Cox regression analysis that incorporated age, along with five single-gene factors, pT-stage, pN-stage, pM-stage, and overall pTNM-stage, to construct a prognostic model (Figure 7A and 7B). The multivariate Cox regression analysis identified FASN, NOTCH3, P4HB, TRIB3, VHL, and pT-stage as independent prognostic biomarkers for BLCA. Additionally, we generated nomograms based on the multivariate Cox regression results to estimate 1-, 3-, and 5-year survival probabilities for individuals with BLCA. Notably, among these biomarkers, VHL exhibited the highest predictive accuracy. Overall, VHL emerged as the most valuable prognostic indicator linked to angiogenesis and stemness in BLCA (Figure 7C). Finally, we created a calibration plot based on the multivariate Cox regression findings, as shown in Figure 7D. This plot illustrates the agreement between predicted and observed survival outcomes, serving as an assessment of the model’s calibration performance.

### VHL interacts with HDAC6 in BLCA

**Figure 8.**
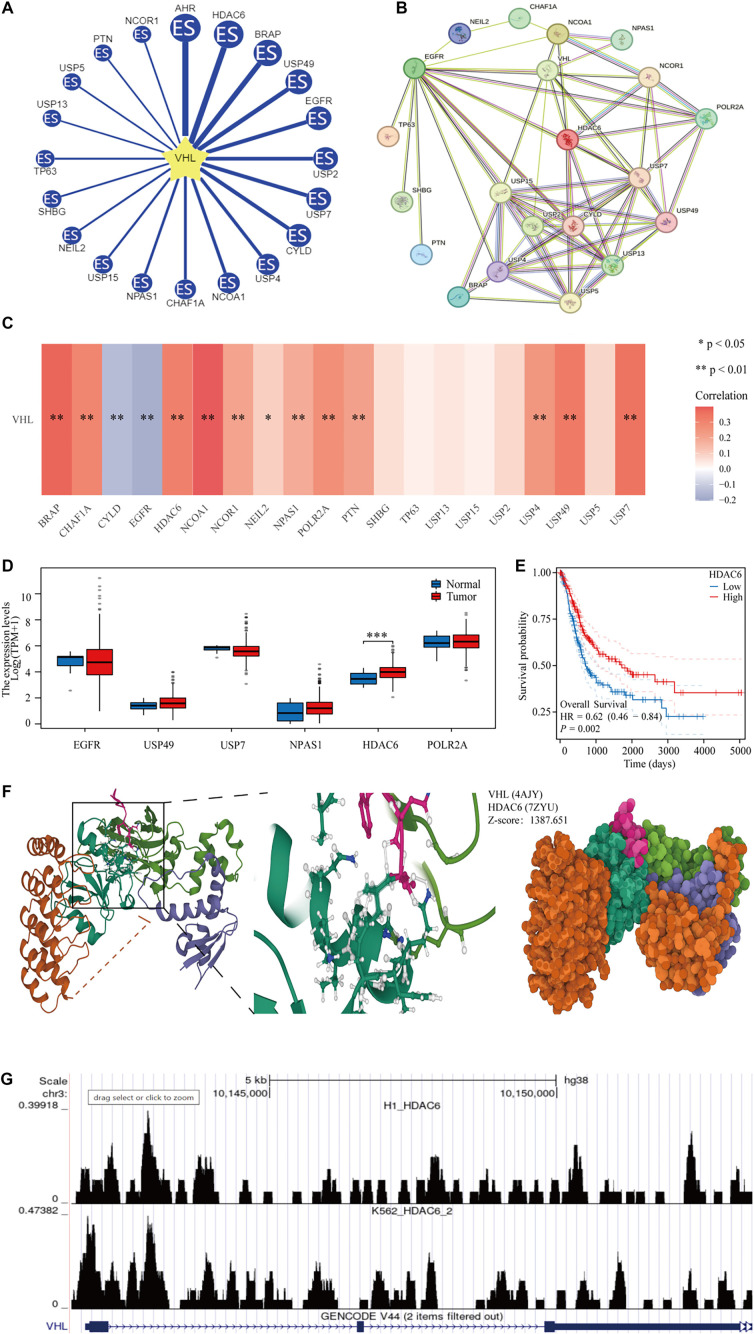
**VHL is highly correlated with HDAC6.** (A) Proteins are predicted to be ubiquitinated by VHL by the UbiBrowser database; (B) Proteins interacting with VHL; (C) Correlation of VHL with its target proteins in the TCGA-BLCA dataset; (D) Differential expression of VHL target proteins in BLCA and paracancerous tissues; (E) KM curves of HDAC6; (F) Docking models of VHL and HDAC6; (G) Analysis of the transcriptional regulation of VHL and HDAC6. VHL: Von Hippel–Lindau; TCGA: The Cancer Genome Atlas; BLCA: Bladder cancer.

VHL has been shown to function as a tumor suppressor across multiple tumor types through its role as an E3 ligase [24, 25]. To identify potential target proteins that VHL may ubiquitinate, we used the UbiBrowser database (Figure 8A). We then investigated interactions between VHL and these proteins using the STRING database (Figure 8B). In the TCGA-BLCA dataset, we identified 14 genes correlated with VHL expression (Figure 8C). Of these, six genes were not only correlated with VHL but also interacted with each other. Among them, HDAC6 exhibited notably different expression levels between BLCA and normal bladder tissues and showed a significant association with BLCA patient outcomes (Figure 8D and 8E). To further explore the relationship between VHL and HDAC6, we conducted a molecular docking analysis, which revealed a structural interaction between the two proteins, yielding a Z-score of 1387.651 (Figure 8F). Furthermore, our analysis of their transcriptional regulatory relationship indicated that HDAC6 regulates VHL transcription.

### VHL is strongly linked to immune infiltration in BLCA

**Figure 9.**
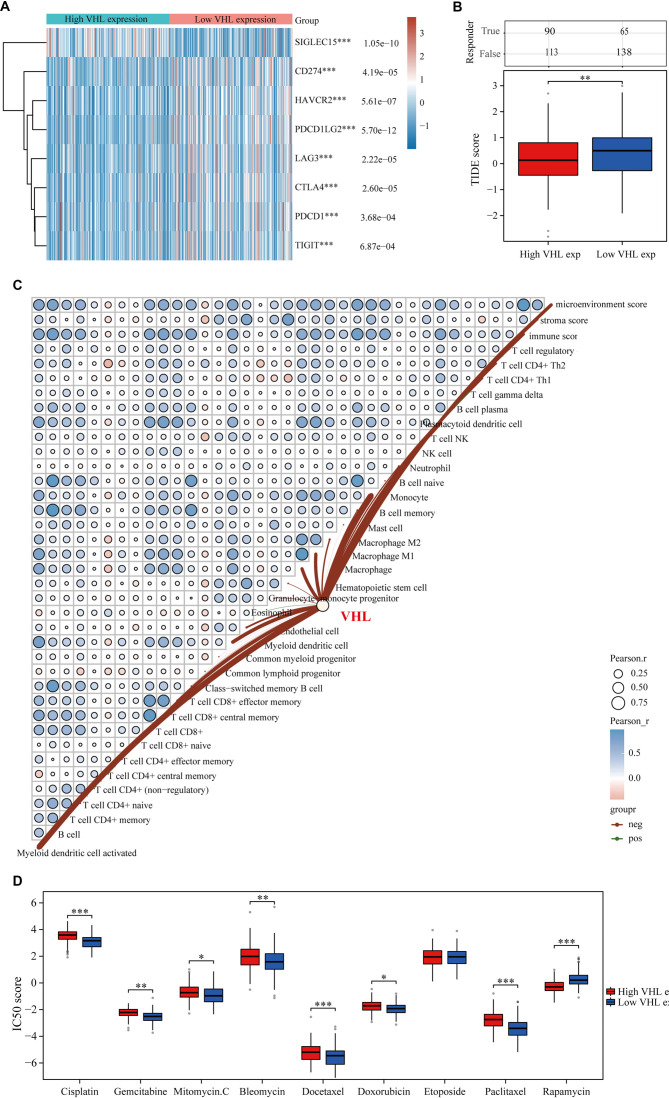
**VHL and BLCA immune microenvironment and chemotherapy sensitivity analysis.** (A) Expression variations of immune checkpoint-associated genes between elevated and reduced VHL expression cohorts; (B) Assessment of anticipated ICI response among elevated and reduced VHL expression cohorts utilizing the TIDE computational model; (C) Examination of VHL associations with immune-infiltrating cellular components through xCELL methodology; (D) Correlation between VHL expression and chemotherapeutic drug sensitivity in BLCA. VHL expression showed significant correlation with the sensitivity to eight chemotherapy drugs, but not with Etoposide. TCGA: The Cancer Genome Atlas; ICI: Immune checkpoint inhibitor; VHL: Von Hippel–Lindau.

The relationship between VHL and immune checkpoint-related genes was initially investigated. Our findings revealed significant differences in the expression of all immune checkpoint-related genes between subgroups with high and low VHL expression (Figure 9A). The high VHL expression group exhibited a notably lower TIDE score compared to the low VHL expression group. Furthermore, patients with low VHL expression had shorter survival following ICB therapy compared to those in the low-risk group (Figure 9B). We then analyzed the association between VHL and immune infiltration-related cells in BLCA using the xCELL method (Figure 9C). Finally, we examined how VHL expression relates to responsiveness to chemotherapeutic agents in BLCA. Results showed that VHL expression was not significantly correlated with sensitivity to Etoposide but was strongly associated with the sensitivity to eight other drugs (Figure 9D).

### Expression and correlation analysis of VHL and HDAC6

**Figure 10.**
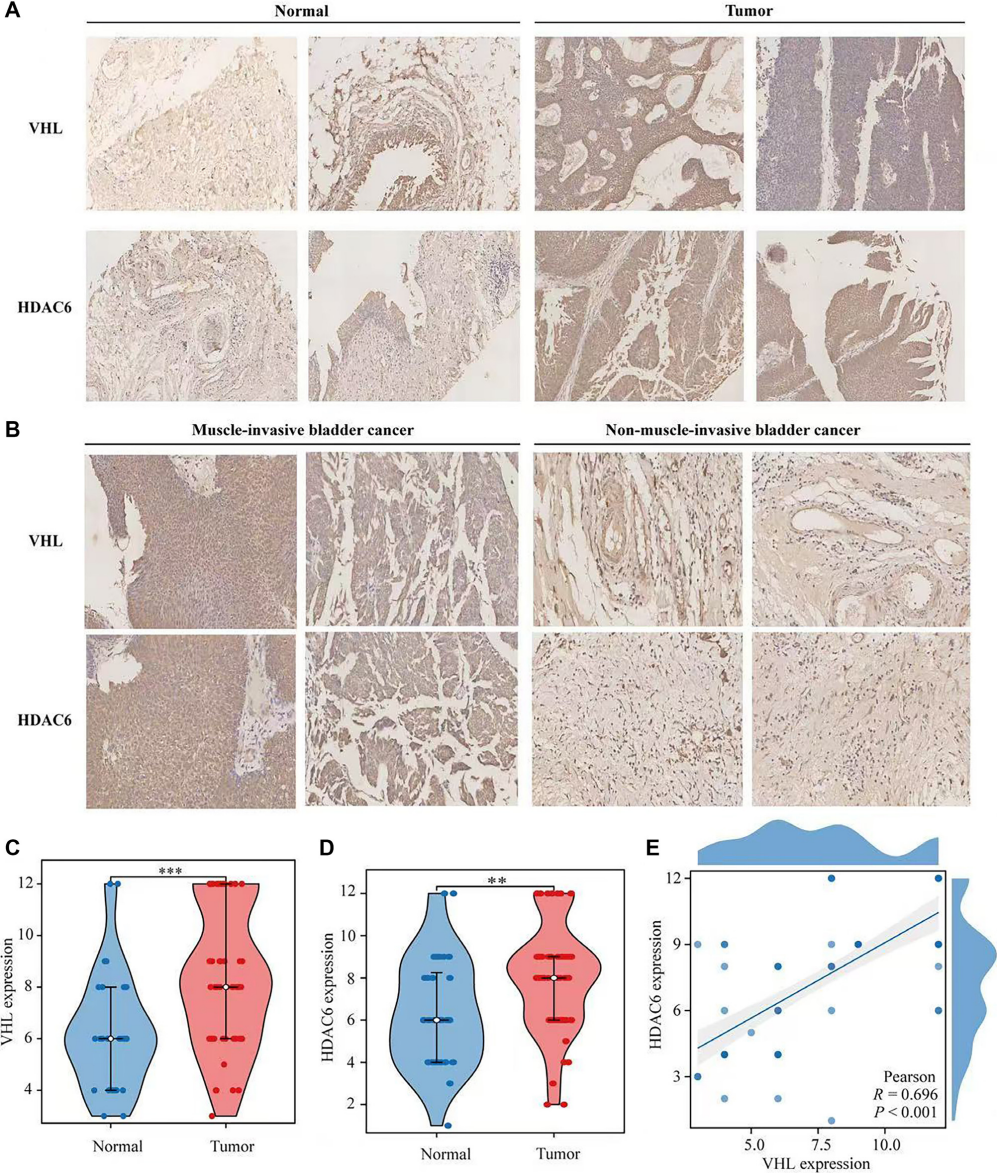
**VHL and HDAC6 are highly expressed and positively correlated in BLCA.** (A) Expression of VHL and HDAC6 in BLCA (×10; The scale: 10µm); (B) Correlation analysis of VHL and HDAC6 in BLCA (×10; The scale: 10µm); (C and D) Violin map of VHL and HDAC6 expression in BLCA; (E) Dot plot of correlation between VHL and HDAC6 in BLCA. VHL: Von Hippel–Lindau; BLCA: Bladder cancer.

To investigate VHL and HDAC6 levels in BLCA, tissue specimens from 40 patients were analyzed using immunohistochemistry. The results showed higher expression levels of both VHL and HDAC6 in BLCA tissues compared to normal bladder tissues (Figure 10A). Correlation analysis further revealed that expression of both proteins was elevated in MIBC compared to non-muscle-invasive cases (Figure 10B). Violin plots illustrating the expression patterns in BLCA and normal tissues are shown in Figure 10C and 10D. Additionally, a dot plot generated via Pearson correlation analysis demonstrated a positive correlation between VHL and HDAC6, with a correlation coefficient of 0.696 (Figure 10E). Together, these findings suggest that VHL and HDAC6 are highly expressed and positively correlated in BLCA, supporting their potential involvement in disease progression.

## Discussion

Cancer is a devastating disease that poses a significant threat to human health and survival [26]. Irregularities in cellular processes contribute to both the initiation and progression of cancer. The unchecked growth of tumors requires a continuous supply of oxygen and essential nutrients [27]. Tumor development is critically dependent on blood vessels, which serve as key conduits for nutrient delivery. However, blood vessels within tumors differ markedly from those in healthy tissues. They often display loose pericyte coverage, disorganized architecture, and excessive dilation. This abnormal vasculature can lead to hypoxic conditions within the tumor mass, hindering the effective delivery of therapeutic agents [28]. To combat this, therapies targeting angiogenesis—aimed at cutting off the tumor’s nutrient supply—have been developed and are currently in use for various tumor types [29, 30]. CSCs retain the ability to self-renew within the tumor microenvironment. Through self-renewal, CSCs contribute to tumor growth by producing more CSCs, while also differentiating into non-CSC tumor cells, promoting tumor heterogeneity and structural complexity within malignant tissues [31]. The processes of blood vessel formation and CSC activity significantly influence tumor recurrence, progression, and metastasis [32]. Moreover, extensive research has revealed a strong correlation between CSCs and tumor vasculature. CSCs have been shown to promote angiogenesis, while a highly vascularized tumor environment supports CSC proliferation [33]. Therefore, a comprehensive exploration of the interplay between angiogenic and stemness traits in BLCA is essential for improving treatment outcomes and patient survival. In this study, we began by analyzing the TCGA-BLCA and GSE13507 datasets to identify differentially expressed genes associated with angiogenesis and stemness that also possess prognostic value. We used the GSCALite platform to assess the significance of these gene sets across various cancer types [34]. While canonical cancer-related pathways—such as TSC/mTOR, RTK, Hormone ER, Hormone AR, EMT, DNA damage response, cell cycle, and apoptosis—are well-established, our analysis found no significant association between P4HB, NOTCH3, and these pathways. Therefore, we focused on examining the relationships between the remaining ten genes and these key pathways. Previous studies have shown that the TSC/mTOR [35, 36] and RTK [37, 38] pathways play critical roles in regulating tumor stemness and angiogenesis. Survival analysis revealed that patients in cluster 1 had worse overall and disease-free survival compared to those in cluster 2. This poorer prognosis may be linked to the activation of IL-17, PI3K-Akt, TNF, P53, NF-kappaB, and HIF-1 signaling pathways, all of which are known to influence tumor angiogenesis and stemness [39–41]. Significant differences between clusters were also observed in terms of immune infiltration in BLCA and responses to immunotherapy. Notably, the IC50 values for standard chemotherapy drugs differed between cluster 1 and cluster 2, suggesting that genes associated with angiogenesis and stemness may influence chemotherapy sensitivity. To construct a prognostic model, we applied the LASSO method, a widely used machine learning technique, to the TCGA-BLCA dataset. The model’s reliability was validated using the GSE13507 dataset. We further examined the relationship between the prognostic model and sensitivity to both immunotherapy and chemotherapy in BLCA. Multifactorial Cox regression analysis identified VHL as the most significant prognostic gene. VHL, an E3 ubiquitin ligase, has been implicated in promoting angiogenesis and stemness by regulating HIF1α activity [42, 43]. Using the UbiBrowser platform, HDAC6 was predicted as a downstream target of VHL, and interaction between the two proteins was supported by STRING database analysis. Molecular docking confirmed a potential binding interaction between VHL and HDAC6. Finally, our study demonstrated a strong association between VHL expression and sensitivity to both immunotherapy and chemotherapy in BLCA.

## Conclusion

We also examined their roles in immune infiltration and the outcomes of both immunotherapy and chemotherapy. Among these genes, VHL emerged as the most significant prognostic indicator. Notably, VHL interacts with HDAC6, which plays a regulatory role in angiogenesis and the expression of stemness markers in BLCA. Furthermore, VHL has shown a strong correlation with treatment outcomes, including responses to immunotherapy and chemotherapy, in BLCA.

## Supplemental data

## Data Availability

The datasets obtained from TCGA database (https://portal.gdc.cancer.gov/) and Genecards database (https://www.genecards.org), GEO database (https://www.ncbi.nlm.nih.gov/geo/), partial analysis by GSCALite website (http://bioinfo.life.hust.edu.cn/web/GSCALite/), STRING (https://string-db.org) database.
